# Supplementary material for: Iron Stress Affects the Growth and Differentiation of Toxoplasma gondii
Source: Int J Mol Sci. 2024 Feb 21;25(5):2493. doi: 10.3390/ijms25052493 (PMC10931281; doi:10.3390/ijms25052493)
Supplement: Supplementary file 1 [file ijms-25-02493-s001.zip › Figures.pdf]

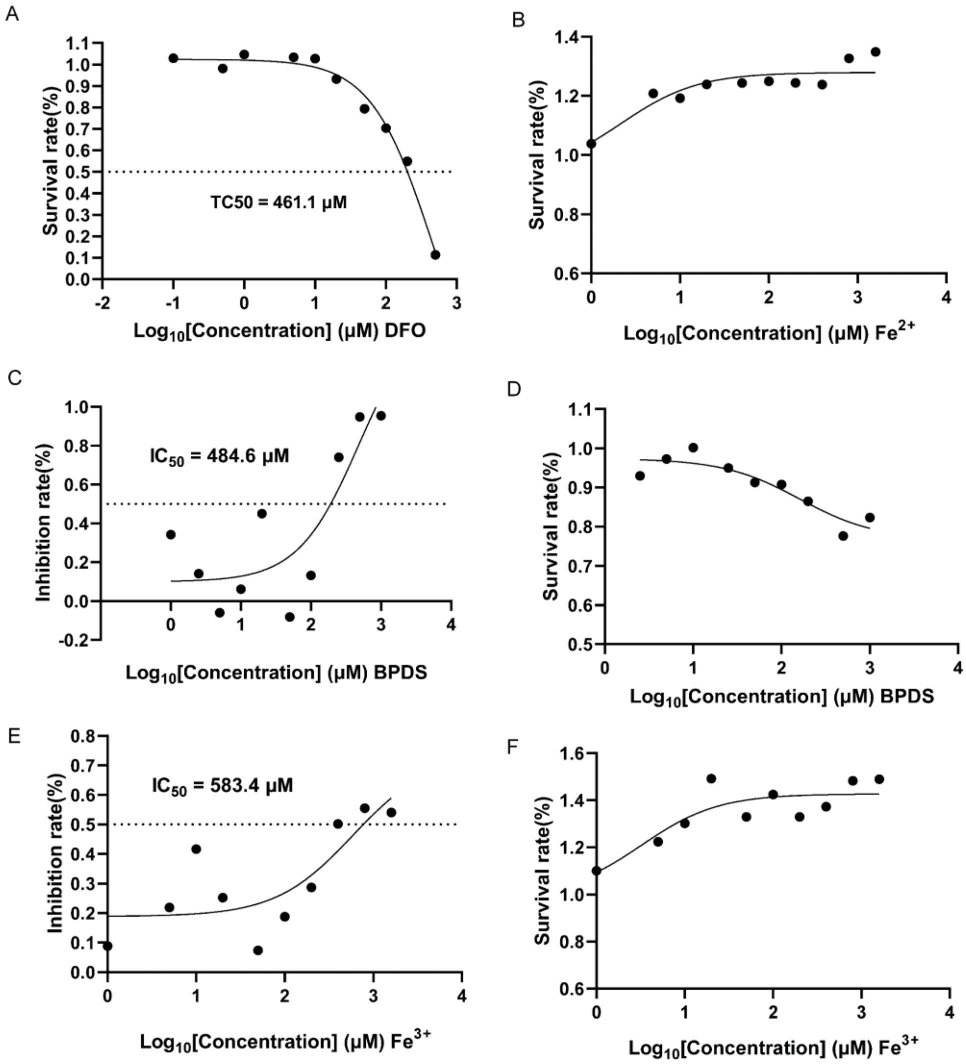

Figure S1

A Cytotoxicity of DFO on HFF cells. Toxicity of DFO at concentrations ranging from 0.1 to 500 μM on HFF cells for 48 h was assessed using the CCK-8 reagent. DMSO was used as a control. The survival rate was calculated as $1- (RLU_{DMSO}-RLU_{DFO})/RLU_{DMSO}$.

B Cytotoxicity of ammonium iron(II) sulfate (1–1600 μM) on HFF cells for 48 h.

C The half lethal concentration curve depicts the effects of BPDS (1–1000 μM) on *Toxoplasma* replication.

D Cytotoxicity of BPDS (2.5–1000 μM) on HFF cells for 48 h.

E The half-lethal concentration curve shows the effects of iron(III) chloride (1–1600 μM) on *Toxoplasma* replication.

F Cytotoxicity of iron(III) chloride (1–1600 μM) on HFF cells for 48 h.

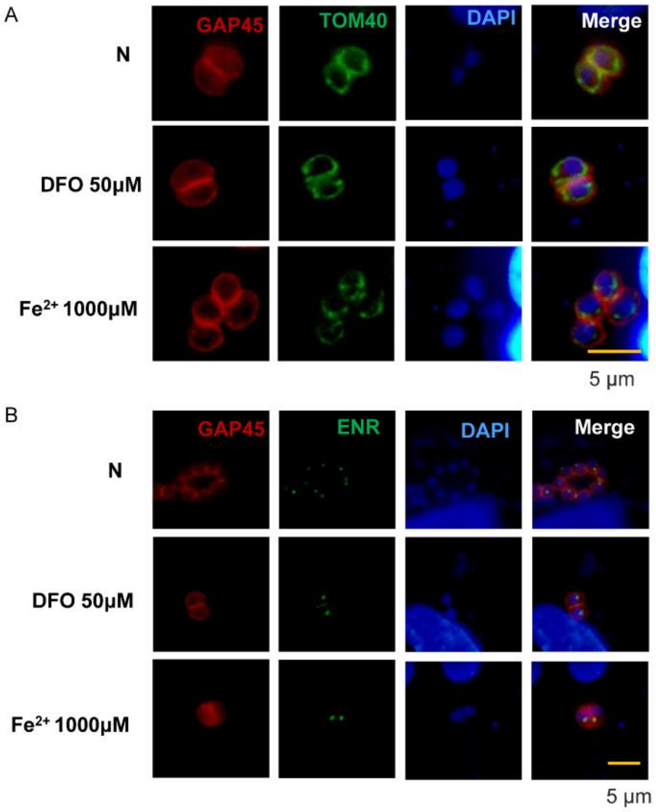

Figure S2

A and **B** Indirect immunofluorescence of *T. gondii* RH tachyzoites under iron depletion. Cells were grown in medium adding with 20 μM DFO, fixed at 24 h post infection and stained with anti-Tom40 (green, **A**) antibody or anti-ENR (green, **B**) antibody, anti-GAP45 (red) antibody, and Hoechst DNA-specific dye (blue). Scale bar, 5 μm.

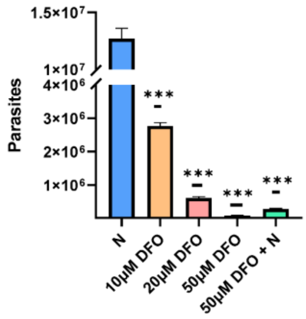

Figure S3

Statistical chart of the number of *Toxoplasma gondii* under different treatment conditions. A total of 1×10^5 tachyzoites were incubated into HFF with a culture medium containing different concentrations of DFO (10 μM, 20 μM and 50 μM) for 72 h, and finally the parasites were isolated by cell scraper and syringe, and were counted. A total of 1×10^5 parasites cultured with 50 μM DFO were placed in normal medium, cultured for 72 hours and counted them.

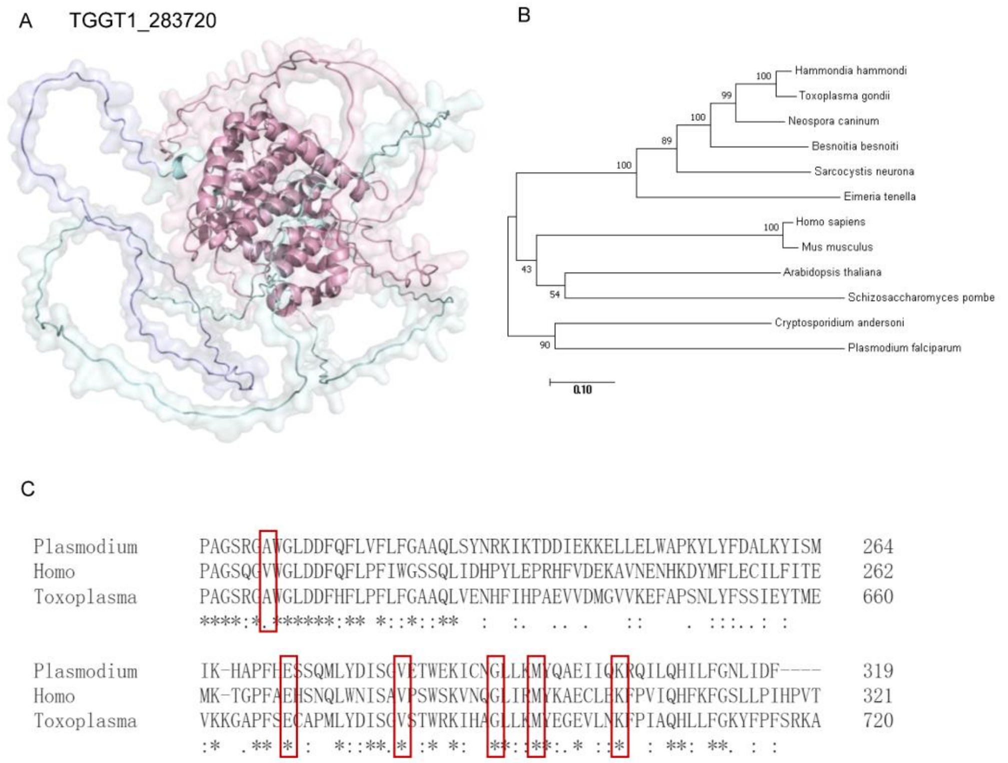

Figure S4

A Protein 3D structures of Toxoplasma gondii PTPA was predicted by AlphaFold2.

B Phylogenetic tree of *Toxoplasma gondii* PTPA protein and PTPA proteins of other organisms.

C Analysis of TgPTPA (TGGT1_283720) amino acid sequence. TgPTPA was aligned with the human PTPA (CAA60163.1) and Pf PTPA (PF3D7_1430100) using ClustalW Multiple Alignment. The identical residues are highlighted with an asterisk, and red frame symbolized amino acids involved in PTPA/PP2A interaction in huma.

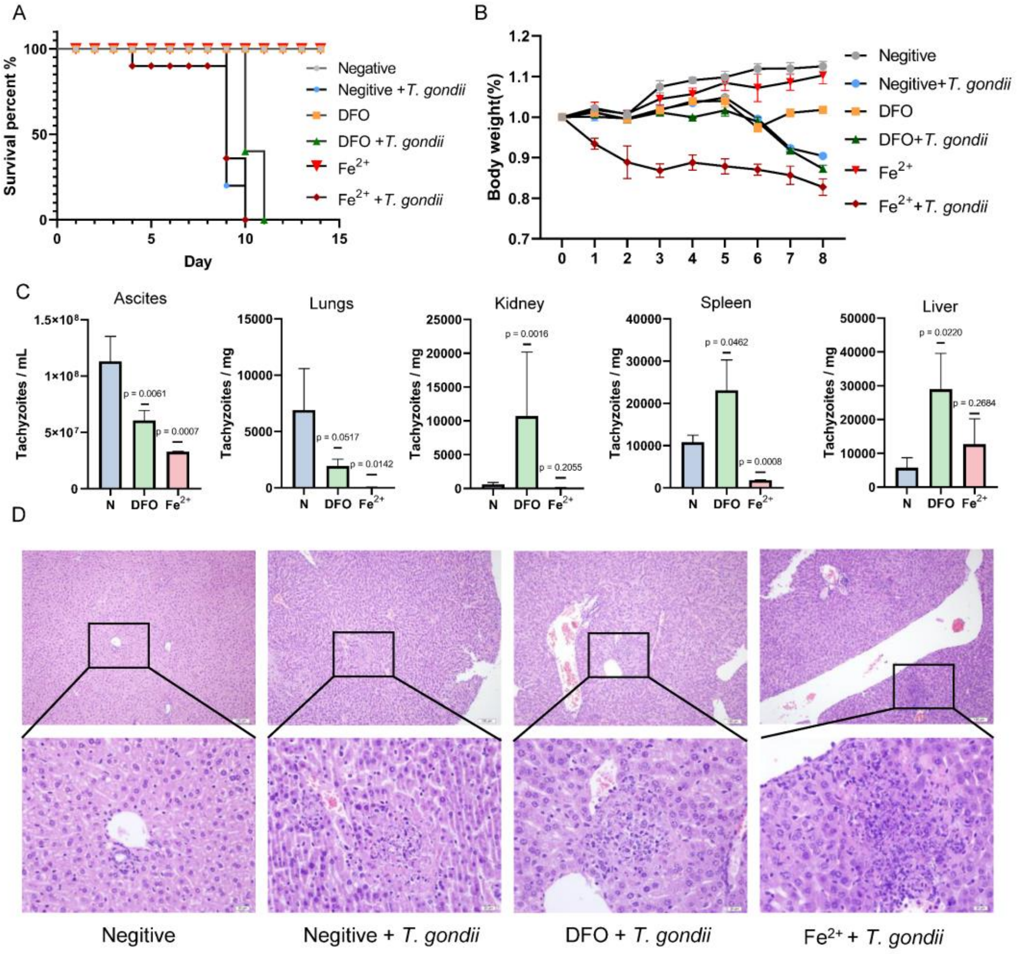

Figure S5 Iron chelation therapy and iron supplementation therapy reveal complex biological processes.

A Survival curve of BALB/c mice intraperitoneally infected with 100 tachyzoites of RHΔku80, treated with iron chelation therapy and iron supplementation therapy. Neither the mice receiving iron chelation therapy nor those receiving iron supplementation therapy showed any change in the mortality rate.

B Weight change of BALB/c mice infected with RHΔku80 , treated with iron chelation therapy and iron supplementation therapy. Iron supplementation in *Toxoplasma*-infected mice resulted in significant weight loss.

C Parasite burden in different organs of infected mice after iron chelation therapy and iron supplementation therapy. Iron chelation treatment reduced the number of *T. gondii* in the peritoneal fluid and lungs of mice, but promoted dissemination and increased parasite burden in other tissues, including the kidneys, spleen, and liver.

D Examination of liver pathological sections showed an increased area of inflammatory necrosis caused by *T. gondii* infection in mice treated with iron supplementation.
